# Overview of Chikungunya Virus Pathogenesis, Genome Variation, Epidemiology, and Control

**DOI:** 10.1016/j.virusres.2026.199703

**Published:** 2026-02-19

**Authors:** Morvarid Hamrahjoo, Faezeh Shams, Nastaran Saadat, Shayan Marhamati, Ali Teimoori

**Affiliations:** a Department of Virology, Faculty of Medicine, Hamadan University of Medical Sciences, Hamadan, Iran; b Department of Microbiology, Faculty of Medicine, Golestan University of Medical Sciences, Gorgan, Iran; c Department of Clinical Biochemistry, Faculty of Medicine, Hamadan University of Medical Sciences, Hamadan, Iran

**Keywords:** Chikungunya, Pathogenesis, Virus receptor, Vaccine, Immunopathogenesis, Epidemiology

## Abstract

Chikungunya virus (CHIKV) is an arthropod-borne alphavirus that causes an abrupt febrile illness typically marked by intense arthralgia, rash, and profound fatigue. Following transmission predominantly through Aedes mosquitoes, the virus is able to infect a broad range of host cell types, such as fibroblasts and macrophages, resulting in acute viremia with high viral titers and, in a subset of patients, prolonged arthritis-like manifestations. CHIKV carries a positive-sense, single-stranded RNA genome that encodes nonstructural proteins essential for viral replication as well as structural components required for virion assembly and host-cell entry. The MXRA8 receptor has recently been identified as a central determinant of viral entry and cellular tropism. Genomic analyses place CHIKV into three major lineages, and certain amino-acid substitutions, most notably the E1-A226V change, have been linked to improved adaptation to Aedes vectors and more efficient transmission. The host immune reaction is multifaceted, involving type I interferons, neutralizing antibodies, and virus-specific T-cell activity. Even so, the virus is able to bypass several of these defenses through established evasion mechanisms, which may contribute to prolonged infection in some cases. Given the continued expansion of Aedes mosquito populations driven by climate change, integrated vector-management strategies remain essential. These include chemical, biological, and genetic approaches aimed at curbing mosquito densities and reducing the impact of insecticide resistance. Sustained global surveillance and coordinated public-health interventions are critical to mitigating the growing burden of CHIKV and addressing the recent surge in large-scale outbreaks.

## Highlights

• MXRA8 identified as pivotal receptor in CHIKV entry.
• Key mutations drive vector adaptation and global spread.
• CHIKV exploits autophagy to enhance viral replication.
• Neutralizing antibodies inform vaccine and therapy design.
• Integrated control strategies crucial for outbreak prevention.

## 1 Introduction and biology

Chikungunya virus (CHIKV) is an arthropod-borne alphavirus that causes abrupt febrile illness, typically marked by severe arthralgia, rash, and fatigue. Transmission occurs predominantly through Aedes mosquitoes, allowing the virus to infect diverse host cell types, including fibroblasts and macrophages, leading to acute viremia and, in some patients, prolonged arthritis-like symptoms. CHIKV carries a positive-sense, single-stranded RNA genome encoding nonstructural proteins essential for replication and structural proteins required for virion assembly and host-cell entry. The MXRA8 receptor has recently been identified as a key determinant of viral entry and cellular tropism (Weaver and Lecuit, 2015).

Chikungunya fever manifests with sudden high fever (often above 39°C), intense muscle and joint pain, headache, fatigue, and rash. Viremia peaks shortly after symptom onset, reaching viral loads up to 10^9 genome copies per milliliter of blood. Unlike dengue, most CHIKV infections are symptomatic. Joint pain is often symmetric, affecting large and small joints, and may involve swelling and arthritis-like inflammation. Rash appears in up to 80 % of cases, typically on the trunk and limbs, with children sometimes developing bullous lesions. Severe complications are rare but can occur in vulnerable populations, including older adults, newborns, and individuals with comorbidities, potentially leading to encephalitis, myocarditis, hepatitis, or multiorgan failure. Chronic or relapsing joint pain resembling rheumatoid arthritis may persist for months or years, significantly impacting patients’ quality of life and socioeconomics. No specific antiviral therapy exists; treatment is primarily supportive (Weaver and Lecuit, 2015).

CHIKV, classified within the *Togaviridae* family and the Alphavirus genus, is known as a progressively important arbovirus in tropical and subtropical regions (Kendall et al., 2019). This family includes the Semliki Forest virus (SFV), Sindbis virus (SINV), Ross River virus (RRV), and Venezuelan equine encephalitis virus (VEEV) (Kendall et al., 2019, Bartholomeeusen et al., 2023). The virus is enveloped, ∼70 nm in diameter, with icosahedral symmetry (Alguridi et al., 2023). Its capsid contains genomic RNA, while surface glycoproteins E1/E2 form trimeric spikes critical for host cell attachment and membrane fusion (de Lima Cavalcanti et al., 2022).

The CHIKV genome is approximately 11,800 nucleotides long and is made up of a positive-sense single-stranded RNA with a 5′ 7-methylguanosine cap and a 3′ poly-A tail. The genome has two open reading frames (ORFs) flanked by the 5′ and 3′ noncoding regions. The first ORF, which covers the first two-thirds of the genome, encodes the nonstructural proteins nsP1, nsP2 (a protease), nsP3, and nsP4 (an RNA-dependent RNA polymerase), all of which play essential roles in assembling the RNA transcription and replication complex (Lozano-Parra et al., 2025). The second ORF, spans 3′ end, encodes subgenomic RNA and codes for structural proteins such the capsid (C) and the envelope glycoproteins E1, E2, E3, and 6 K. These proteins are involved in viral particle assembly and entry into host cells (Yap et al., 2017). E1 and E2 mediate viral binding and fusion, while E3 and 6 K contribute to particle maturation. Viral polyproteins are processed into mature structural proteins, assembling into infectious virions (Yap et al., 2017, Wang et al., 2024).

The replication of CHIKV mainly has the same patterns as other alphaviruses, although there are still many special biological aspects that are not completely understood. CHIKV enters the human body primarily through the bite of infected *Aedes mosquitoes* (Hoornweg et al., 2016). This virus is capable of infecting a wide array of vertebrate and invertebrate cellular entities, presumably utilizing glycosaminoglycans as attachment factor rather than relying on particular protein receptors. For many years, the primary receptor of CHIKV remained unknown, and several host factors were proposed as potential attachment factors or co-receptors. The finding that the protein matrix remodeling associated (MXRA8) acts as the main entry point for CHIKV and other alphaviruses linked to arthritis marks a notable breakthrough (Zhang et al., 2018). This aligns with the virus's tendency to infect joints, muscles, and related tissues. MXRA8 is a well-preserved transmembrane protein found on crucial target cells for CHIKV, including fibroblasts, osteoblasts, and muscle cells (Feng et al., 2023). Research into the structure has shown that the E2 envelope glycoprotein of CHIKV attaches itself to particular regions on MXRA8 with great strength (Rahman et al., 2025).

Alternative factors, including the Prohibitin (PHB) complex and CD147, may assist entry in some cell types but are less critical than MXRA8 (Wintachai et al., 2012). The virus mostly enters host cells by clathrin-mediated endocytosis, while the acidification of the endosome triggers the fusion of the viral envelope with the host cellular membrane, consequently releasing viral RNA into the cytoplasmic compartment (Reyes Ballista et al., 2023). Following entry, the genomic RNA is translated into nonstructural proteins forming the replicase complex, which synthesizes negative-sense RNA within protective spherules. These evolve into cytopathic vacuoles (CPV-I), and polyprotein processing shifts to produce positive-sense genomic and subgenomic RNAs (Laurent et al., 2022, Girard et al., 2024).

Subgenomic RNA encodes structural proteins that assemble into nucleocapsids and glycoproteins, maturing through the host secretory pathway. Viral budding occurs at the plasma membrane, assisted by glycoprotein complexes, with CPV-II structures potentially serving as assembly intermediates (Girard et al., 2024).

CHIKV infection triggers various host antiviral responses, including apoptosis and interferon production. However, viral proteins nsP2 and nsP3 help counter these defenses, and autophagy appears to promote viral replication by limiting cell death. Although some host factors involved in CHIKV replication are known, detailed cellular mechanisms are still being explored. Understanding CHIKV’s replication in both mammalian and mosquito hosts is essential for developing antiviral strategies (McAllister et al., 2020).

## 2 Genetic diversity of CHIKV

CHIKV shows significant genetic and lineage-level diversity across different regions of the world, but serotypically it consists of only one serotype, indicating cross-protection among different genotypes (Sumathy and Ella, 2012).

Phylogenetic studies have shown that the CHIKV comprises three major genotypes or lineages: West African (WA), East-Central-Southern African (ECSA), and Asian. In addition, the Indian Ocean lineage (IOL) has been identified as a subclade of the ECSA genotype.These genotypes have different epidemiological characteristics, including variations in their ability to be transmitted by the two primary mosquito vectors Aedes aegypti and Aedes albopictus (Sharif et al., 2021, Chen et al., 2016). Most post-2006 strains belong to the ECSA lineage, particularly the IOL subclade, which is associated with the key E1-A226V mutation in the glycoprotein and has increased compatibility with the vector Aedes albopictus. In India, two ECSA subclades have been identified that align with the diversity of local vectors, and still, only a single serotype has been maintained (Sumathy and Ella, 2012, Sharif et al., 2021). After the introduction of the virus in 2014, both the ECSA and Asian/Caribbean lineages were identified simultaneously. The ECSA genotype, which was initially introduced from Bahia (Brazil), is now predominant throughout the country, and a new American subtype is circulating (De Souza et al., 2024, Resck et al., 2024). Global genomic analyses have shown that most genetic changes occur in the E1 and E2 genes, which play key roles in the virus’s adaptation to different vectors. A combination of mutation, recombination, and occasional co-infection between Asian and ECSA lineages has contributed to the emergence of considerable genetic diversity in certain region (Ning et al., 2024).

While the Asian genotype mostly adapts to Aedes aegypti rather than Aedes albopictus, it occasionally shares compensatory or vector-adaptive modifications like E1-A226V, though less frequently than ECSA/IOL strains. Some Asian strains that co-circulate with ECSA have been shown carrying E2-L210Q, which accelerates the transmission of albopictus during hybrid outbreaks. Changes in non-structural proteins (nsPs), such as those that impact replication (such nsP2 or nsP3 variations), exist among all genotypes but can be less harmful in Asian lineages (Lanciotti and Valadere, 2014, Jatin Shrinet et al., 2012). Asian genotype strains exhibit distinct E1 and E2 modifications, like E1-V226A (reversal of ECSA's A226V) for aegypti optimization and E2-P318S or E1-I317M, that are not prevalent in ECSA. Apart from ECSA's E1-K211E/E2-V264A pair, Asian/Caribbean subclades in India and the Americas present new E1-K211T or E2-L210 variations.In compared to ECSA's IOL-focused adaptations, recombination hotspots in E1/E2 junctions produce Asian-specific variants (Jatin Shrinet et al., 2012, Chen et al., 2021).

## 3 The pathogenesis of CHIKV

The common reservoirs of CHIKV are monkeys and other vertebrates. The role of cattle and rodents has also been reported in the transmission of this virus (Presti et al., 2014). The incubation period of chikungunya virus typically ranges from 2 to 12 days after the bite of an infected mosquito (Rudolph et al., 2014). Most people show symptoms 3 to 7 days soon after getting infected (Ojeda Rodriguez and Walker, 2023). Based on 204 observations from 35 studies; they realized that an average incubation period was 5.9 days and that 95 % of the people developed symptoms between 3.4 and 10 days (Rudolph et al., 2014). The incubation period varies based on factors ranging from viral load and the immune response of the host (Ojeda Rodriguez and Walker, 2023). Dermal fibroblasts, keratinocytes, endothelial cells, and resident macrophages as the primary infection sites (Gasque et al., 2015). In along with these, dendritic cells in lymph nodes become infected, which helps the virus spread to the bloodstream through the lymphatic system (de Lima Cavalcanti et al., 2022).

Glycosaminoglycans are the primary molecules that allows viruses attach to host cells (Silva and Dermody, 2017). They do this by facilitating the virus get in by clathrin-mediated endocytosis (Silva and Dermody, 2017). After the endosome becomes acidic, the viral and host membranes merge, letting viral RNA into the cytoplasm so they can replicate (Silva and Dermody, 2017). The virus then forms multiple copies of it before spreading to the lymph nodes and into the bloodstream, enabling systemic dissemination (Ganesan et al., 2017). After infecting synovial fibroblasts, macrophages, myocytes, and osteoblasts in musculoskeletal tissues, the virus causes damage to cells through causing apoptosis and pyroptosis, which sets off inflammatory cascades (de Souza et al., 2024). Joint pain, swelling, occasionally chronic arthritis that persist for months to years after infection are caused by an interaction of immune-mediated mechanisms and direct viral cytopathic effects (Amaral et al., 2023). Chronic inflammation and joint degradation are sustained by viral persistence in synovial macrophages and fibroblasts, which leads to in the prolonged production of inflammatory mediators such as IL-6, IL-8, and matrix metalloproteinases (Amaral et al., 2023).

According to a recent study, CHIKV can get able to improve replication while suppressing immune surveillance through taking advantage of host cell autophagy pathways (de Souza et al., 2024). CHIKV first triggers autophagy by stimulating the IRE1α–XBP-1 signaling pathway and blocking mTOR, which is a significant negative regulator of autophagy. This occurs when stress is put on the endoplasmic reticulum (ER) and oxidative stress pathways by virus replication into ER. This early onset of autophagy diminish apoptosis in cells that are infected, which allows them live longer and spread the virus more rapidly. Research findings using inhibitory drugs (e.g., 3-Methyladenine) or genetic silencing of autophagy-related genes (e.g., Beclin-1) resulted in decreasing viral replication, demonstrating that CHIKV modulates autophagy for effective replication (Krejbich-Trotot et al., 2011).

CHIKV infection of the vascular endothelium may cause changes in vascular permeability that are thought to contribute to the rare instances of hemorrhagic manifestations seen in some patients. CHIKV infection leads to an increase in reactive oxygen species (ROS) and superoxide anions. These molecules are highly reactive and cause oxidative stress, which directly damages the walls of the blood vessels. Moreover, the infection triggers the release of a variety of pro- and anti-inflammatory cytokines. This shift in the biochemical balance within the vessels further contributes to their disruption. follow infects the endothelial cells, it causes them to increase the expression of several key molecules like Adhesion Molecules (ICAM-1) that are typically involved in immune cell recruitment but, when overexpressed, contribute to the activation and dysfunction of the endothelium. Likewise, Inducible Nitric Oxide Synthase (iNOS) is responsible for the massive production of nitric oxide (NO). While NO plays roles in normal vessel function, its overproduction acts as a signaling molecule that, along with the other changes, induces endothelial activation and destabilizes the endothelial barrier's integrity (Oliveira-Neto et al., 2024).

The infection also causes matrix metalloproteinases (MMPs), especially MMP-9, to be upregulated (Ganesan et al., 2017). These types of enzymes break down tight junction proteins such as claudin and occludin, that makes endothelial junctions more fragile while making them become more permeable (Ganesan et al., 2017). These chemical changes cause plasma and immune cells to leak into tissues, which may contribute to edema, petechiae, and in certain cases, severe hemorrhage Clinically, these complications increase morbidity and may result in hypotension and shock, requiring supportive medical care for managing fluid imbalance (Loaiza-Cano et al., 2024). Furthermore, destroying the vascular barrier makes it easier for viruses to spread resulting in the inflammatory responses caused by with chikungunya infection worse, which are often painful in the joints and muscles (Amaral et al., 2023, Kril et al., 2021). Elevated acute-phase proteins, pro-inflammatory cytokines, mild thrombocytopenia, and temporary coagulation abnormalities attributed to systemic inflammation are commonly observed in laboratory results of CHIKV infection (Gasque et al., 2015, de Lima Cavalcanti et al., 2022). In addition, blood, saliva, urine, and cerebrospinal fluid may contain infectious viral particles or RNA, which suggests a variety of viral shedding pathways (de Lima Cavalcanti et al., 2022).

The virus modulates macrophage responses to evade immune clearance and promote survival (Guerrero-Arguero et al., 2020). Transcriptome analysis show that CHIKV infection changes important signaling pathways in macrophages, which could be a good target for treatment (Gray et al., 2022). Additionally, macrophages contain replicating viral RNA in joint tissues, which leads to long-term inflammation and pain in the joints (Zarrella et al., 2025). Her et al., investigates the role of blood leukocytes, specifically monocytes, during the acute phase of chikungunya virus (CHIKV) infection. They have shown infection of monocytes during the acute, early phase of infection is significant because these cells, which differentiate into macrophages, are later found in the synovial tissues of chronically infected patients. They also detected CHIKV antigens, an indicator of active viral replication, were primarily detected in monocytes (CD14+). Furthermore, was a clear and significant correlation between the patient's blood viral load and the percentage of CHIKV Ag-positive monocytes. Patients with high viral loads had a significantly higher percentage of infected monocyte (Her et al., 2010).

## 4 Immune response to the CHIKV

Upon CHIKV infection, innate immunity is promptly activated by endosomal Toll-like receptors (TLR3, TLR7, and TLR8) and cytoplasmic sensors, such as RIG-I and MDA5, which can detect viral RNA and start signaling cascades that generate type I interferons (IFN-α and IFN-β) (Fox and Diamond, 2016). However CHIKV may also disrupt TLR signaling pathways in order to prevent innate immune responses. The viral non-structural protein 2 (nsP2) hinders both Toll-like receptor (TLR) and RIG-I-like receptor (RLR) pathways by blocking key signaling proteins, like TBK1 and IRF3, as being essential for interferon production (Raj et al., 2025). This inhibition affects the downstream regions of the TLR signaling pathway, resulting in challenging to develop type I interferons and Interferon stimulating genes (ISGs) (Raj et al., 2025). In addition, while CHIKV infection initially increases the expression of TLR3, TLR7, and TLR8, persistent infection blocks interferon responses, likely through nsP2-mediated interference (Valdés-López and Fernandez, 2022).

A number of studies indicates TLR4 assists in the attachment and entry of CHIKV via envelope protein E2, but is not active in following stages of viral replication (Mahish et al., 2023). Pharmacological suppression of TLR4 reduces viral load and pro-inflammatory cytokines. Generally, CHIKV targets signaling molecules located downstream of TLRs to reduce the viral defense and help the virus persist in its host system (Kayesh et al., 2025). TLR3 plays an important role in controlling CHIKV infection, replication, and pathology. It recognizes the double-stranded RNA replication intermediate of the virus. Studies in mice and human fibroblasts with defective TLR3 signaling have shown markedly increased viral replication and more severe pathology, indicating that TLR3 normally plays a protective, antiviral role (Her et al., 2015).

Interferon-stimulated genes (ISGs), that restrict viral replication and enhance antigen presentation, are triggered by these interferons (de Lima Cavalcanti et al., 2022). To interfere with antiviral signaling, CHIKV uses various types of evasion strategies, including hindering TBK1 activation and blocking STAT1 phosphorylation (de Souza et al., 2024). Several types of ISGs were shown to himder CHIKV replication in different mechanisms. ISG15 acts as an immunomodulator during CHIKV infection. Mice lacking in ISG15 demonstrate increased susceptibility and higher proinflammatory cytokine levels, despite unaffected viral loads, which suggests that the ISG15 functions to regulate inflammation rather than having a direct antiviral effect (Werneke et al., 2011). The IFIT family (IFIT1, IFIT2, IFIT3, IFIT5) inhibits viral RNA from being translated and replication by binding to viral RNA that lacks the right modifications (White et al., 2011). Viperin makes ddhCTP, a ribonucleotide analog that disrupts CHIKV RNA synthesis and subsequently hinders viral replication (Carissimo et al., 2019). ISG20 is an exonuclease that degrades viral RNA and indirectly regulates the replication of CHIKV viral RNA (Weiss Christopher et al., 2018). CHIKV blocks these antiviral ISGs by preventing the phosphorylation of STAT1 and nuclear translocation. It inhibits interferon signaling and the synthesis of downstream ISGs (Fros et al., 2010).

Controlling CHIKV infection is mainly dependent on humoral immunity; neutralizing IgM and IgG antibodies against E1 and E2 envelope glycoproteins are crucial for viral clearance and avoiding reinfection (de Lima Cavalcanti et al., 2022). The linear epitope E2EP3, located at the N-terminus of E2, comparises up to 80 % of early neutralizing IgG and is notably exposed on the viral surface, leading to protective responses that reduce viremia and joint inflammation (Kam et al., 2012). Moreover, the acid-sensitive region surrounding residue 162, crucial to viral fusion, and the N218 residue, which IgM antibodies target to disrupt viral attachment, function as two other critical neutralizing sites (Selvarajah et al., 2013, Lam et al., 2015). These neutralizing epitopes are mainly found on the membrane-distal domain A of E2, which is accessible on the viral spike. E1 glycoprotein epitopes also contribute neutralizing antibodies but are less predominant. These specific epitopes are essential for vaccine development and therapeutic antibody synthesis designed to targeting CHIKV (Fong et al., 2014). These antibodies mediate antibody-dependent cellular cytotoxicity (ADCC) preventing viral entry.

Jin et al. demonstrate that neutralizing antibodies against CHIKV not only block the virus in entering cells, but they induce antibody-dependent cellular cytotoxicity (ADCC) against cells that are already infected. In their study, using advanced microscopy and functional assays, they found that these antibodies crosslink viral glycoproteins on the surface of infected cells, hence activating Fcγ receptors on immune effector cells, notably natural killer (NK) cells, resulting in ADCC and the removal of infected cells (Jin et al., 2018). This process depends on the concentration which required Fc glycosylation to bind to the Fcγ receptor. Another study indicated that vaccinations against CHIKV make antibodies that assist NK cells degranulate and release cytokines. This further supports the concept that ADCC is critical for protective immunity (Tschismarov et al., 2021).

Infection-induced memory B cells provide long-term humoral immunity and can persist for years; they tend to be detected over ten years after infection (Fox and Diamond, 2016). While CD8+ cytotoxic T lymphocytes degrade infected cells in order to prevent the spreading of the viral infection, CD4+ T cells support B cells to coordinate cytokine responses (Amaral et al., 2023). (Dias et al., 2018). CTLsdirectly killing infected cells through cytolytic mechanisms which include release of cytotoxic granules that contain granzyme B and perforin, and by using death receptors such CD95/CD95L (Dias et al., 2018). Activated CD8+ T cells produce antiviral cytokines which help the immune system resist to viral infections (Davenport et al., 2020). Studies showed expression of CD107A (a degranulation marker), granzyme B, and perforin in CD8+ T cells from patients in the acute phase of CHIKV infection was increased, suggesting severe cytotoxic responses.

CD8+ T cells develop in infected tissues and conduct their function as receptors, but they do not appear to be efficient at removal of CHIKV from joint tissues (Davenport Bennett et al., 2020). This might be due to the virus has mechanisms for escape from the CTLs . On the other hand, CD4+ T cells promote B cell maturation and antibody formation by releasing essential cytokines, thereby coordinating a strong immune system response (Poh et al., 2020). For effective viral clearance and disease resolution, CD8+ cytotoxic activity and CD4+ T cell-mediated support have to function simultaneously (Poh et al., 2020). Though reinfection memory T cells, especially the central and effector memory subsets, can last for years and adjust urgently to recall (Fox and Diamond, 2016).

IL-6, IL-1β, TNF-α, IFN-γ, and chemokines such as CXCL10 and CCL2 are pro-inflammatory cytokines that have been significantly increased in CHIKV infection. These cytokines promote the recruitment of immune cells, but if unregulated, it can also damage tissue (de Lima Cavalcanti et al., 2022). In order to diminish tissue damage and inflammation, regulatory cytokines like IL-10 are also upregulated (Fox and Diamond, 2016). In comparison with monocytes and macrophages, which contribute to both viral clearance and inflammation by shedding reactive oxygen species and matrix-degrading enzymes, natural killer (NK) cells act early by producing IFN-γ and directly killing infected cells (Fox and Diamond, 2016).

Clinical outcomes, ranging from mild fever illness to severe chronic arthritis, are characterized by the balance between immunopathology and immune protection, which is influenced by host genetics and viral factors (Amaral et al., 2023). Also, new evidence highlights to the emerging roles of inflammasome activation and mitochondrial antiviral signaling pathways (MAVS) in CHIKV pathogenesis and immunity. It may be feasible to mitigate the severity of the disease by therapeutically targeting these pathways (de Souza et al., 2024). Inflammasomes are complexes of multiple proteins in the innate immune system that detect infections or stress in cells and activate on caspase-1. The enzyme converts proinflammatory cytokines IL-1β and IL-18 into active forms which promote inflammation (Gomes de Azevedo-Quintanilha et al., 2022). In CHIKV infection, the NLRP3 inflammasome is activated in immune cells, such as platelets and macrophages, which leads to the release of IL-1β, that worsens inflammation and results in joint pain and tissue damage (Gomes de Azevedo-Quintanilha et al., 2022). Viral replication triggers these inflammasome sensors, which serve as indicators for determining how severe the disease is. Targeting inflammasome activation or its cytokine products offers a possible therapeutic strategy for reducing CHIKV disease severity (Shi et al., 2023).

## 5 Asymptomatic CHIKV infection

When relying solely on clinically apparent cases, these asymptomatic patients may still develop detectable viremia at levels adequate to infect mosquitoes, raising concerns about their potential contribution to further transmission and underestimate the true force of infection (Skalinski et al., 2023). Early outbreak investigations and transfusion-risk assessments estimated that roughly 3–28 % of infected persons remain asymptomatic, indicating that a substantial minority of infections may go unrecognized during epidemics (Nakkhara et al., 2013). More recent cohort and cluster studies have reported even higher proportions of inapparent infection in some settings; for example, a Nicaraguan household-based study found that nearly half of laboratory-confirmed infections were asymptomatic, highlighting that the asymptomatic-to-symptomatic ratio can vary widely by population and viral lineage (Appassakij et al., 2013). These asymptomatic individuals may still develop detectable viremia at levels sufficient to infect mosquitoes, raising concerns about their potential contribution to ongoing transmission and to underestimation of the true force of infection when relying solely on clinically apparent cases (Bustos Carrillo et al., 2019). Furthermore, systematic reviews of CHIKV seroprevalence indicate substantial cumulative infection rates in many endemic areas, implying that surveillance systems focused on overt febrile arthralgia will miss a proportion of infections and may underestimate both the population immunity and the overall disease burden (Appassakij et al., 2013). although CHIKV is generally considered a highly symptomatic arbovirus, the documented range of asymptomatic infection has important implications for outbreak detection, transfusion safety, and the interpretation of seroepidemiological studies (Skalinski et al., 2023).

While viremic donors without symptoms can pass on the virus through blood products, asymptomatic CHIKV infections among blood donors could pose a serious public health and transfusion safety threat (Ngwe Tun et al., 2023). CHIKV RNA has been detected in asymptomatic blood donors in Brazilian studies performed during recent outbreaks, suggesting that usual symptom-based deferral rules fail to spot people with infection within the viremic window (Giménez-Richarte et al., 2022). The variation between clinical screening and virologic reality was highlighted in one such investigation when molecular screening identified recent viral infection with CHIKV in a subset of healthy donors who failed to report any recent severe arthralgia (Ngwe Tun et al., 2023). Similarly, research from endemic regions in india documented CHIKV IgM positivity and elevated viremia in asymptomatic donors, confirming that silent infections occur at measurable rates during epidemics (Verma et al., 2021). A comprehensive evaluation of arboviral threats further assumes that 1–6 % of donors could contain CHIKV during peak transmission, mostly without overt symptoms, across many countries including Myanmar, Puerto Rico, and Latin America (Ngwe Tun et al., 2023, Kyaw et al., 2020). In an attempt to mitigate transfusion-transmitted dangers during CHIKV outbreaks, these findings from various geographic locations highlight the importance of nucleic acid testing or increased surveillance in blood banks (Ngwe Tun et al., 2023, Kyaw et al., 2020).

## 6 CHIKV vaccines

By 2025, several kinds of vaccines have been developed to protect humans against CHIKV infection, and two of the strongest candidates are close to the regulatory approval stage (Weber et al., 2024, ANON, 2026a). VLA1553 (IXCHIQ), a live-attenuated CHIKV vaccine developed by Valneva SE, is the first licensed vaccine (Weber et al., 2024). In November 2023, the FDA approved it for use in those who were 18 years of age or older and at high risk of becoming infected with CHIKV (Hills et al., 2025). In 2024, it was also approved in Canada and the EU (Hills et al., 2025). The FDA suspended IXCHIQ's use in August 2025 pending further safety review due to a single intramuscular dose of the medicine creates strong neutralizing antibody responses, recent post-marketing surveillance has shown safety concerns, including chikungunya -like symptoms, hospitalizations, and rare fatalities (Weber et al., 2024, ANON, 2026b).

In Phase III clinical trials, a second advanced candidate, PXVX0317 (Vimkunya), a virus-like particle (VLP) vaccine, showed high immunogenicity and safety, achieving seroconversion rates above 97 % within 22 days after vaccination (Weber et al., 2024, Richardson et al., 2025). The FDA and European Medicines Agency (EMA) are currently providing it priority review, and approval anticipated in 2025 (Weber et al., 2024).

In order to produce neutralizing antibodies that inhibit viral entry and replication, both vaccines target the structural envelope proteins E1 and E2 (Weber et al., 2024). Long-term protection, efficacy in endemic populations, and safety in particular groups such as pregnant and immunocompromised individuals are being studied by ongoing Phase IV and real-world research. The development of CHIKV vaccines signifies an important development in the control of a disease with a high globe burden, despite the impediments with IXCHIQ (Ribeiro Dos Santos et al., 2025).

Adults between the ages of 18 and 64 who intend to travel to areas with active CHIKV transmission or recent outbreaks are now advised by the CDC to consider immunization, particularly for stays longer than two weeks that involve mosquito exposure. Since modeling indicates that vaccinations could prevent 70–90 % of chronic post-CHIKV arthralgia cases despite 10–20 % reactogenicity rates, we support targeted vaccination for these high-risk travelers and epidemic responders where exposure risk is significant (Schwameis et al., 2016). For older persons or short-term low-risk tourists (less than two weeks), routine immunization is not recommended until further safety data on uncommon neurologic adverse effects following licensure are obtained.Targeted mass vaccination programs among adults should be considered in conjunction with increased vector control measures in hyperendemic regions where outbreaks are explosive. Individual risk variables, vaccine reactogenicity profiles, and area transmission intensity should all be considered when making vaccination decisions through shared clinical counseling.

## 7 Diagnosis

Commercial immunochromatographic test (ICT) kits for chikungunya virus (CHIKV) remain scarce, mostly targeting antibodies (IgM/IgG) with minimal antigen-detection alternatives, which limits effective point-of-care testing (Andrew et al., 2022). Due to slow IgM seroconversion and decreased viremia during early acute infection, IgM-based fast ICT kits show low overall pooled sensitivity at 42.3 %, falling to 26.2 % for samples taken ≤7 days following symptom onset. Commercial antigen-detecting ICTs are uncommon and differ by genotype (e.g., decreased efficacy against Asian lineage), despite the fact that they reach higher acute-phase sensitivity (82–86 %, occasionally 100 % in prototypes). ICTs are insufficient for independent acute-phase diagnosis due to the better sensitivity of IgM ELISA kits (93.4 %) (Andrew et al., 2022, Burdino et al., 2016). Although antibody ICTs have a high specificity (96–97 %), they are susceptible to cross-reactivity with dengue or Zika viruses because of similar clinical symptoms, which can result in false positives in endemic co-circulation zones; multiplex tests provide some mitigation but are still limited 94–96 % specificity across lineages is provided by antigen ICTs; however, thorough validation is not available (Moreira et al., 2022, Chusri et al., 2015, Guo et al., 2025). The market is disjointed (over 43 antibody ICTs, predominantly from Brazil, USA, and India; 23 approved by ANVISA, none by EMA or FDA); Phase IV real-world impact evaluations are absent, with most studies limited to biased Phase I/II case-control designs. Promising in-house antigen prototypes exist but lack commercialization; fulfillment of REASSURED standards (affordable, stable, equipment-free) is essential for practical deployment (Andrew et al., 2022, Moreira et al., 2022).

## 8 CHIKV cycle in mosquito

After taking a viremic blood meal, CHIKV enters the mosquito midgut lumen and infects midgut epithelial cells (Johnson et al., 2023), in the midgut epithelium *Aedes aegypti* and *Aedes albopictus*, CHIKV replicates locally, for the virus to disseminate beyond the midgut, it must cross the “midgut escape barrier” (MEB) — the basal lamina underlying the midgut epithelium and other tissue barriers, viral replication kinetics (e.g., high replication rate) and viral adaptations facilitate escape from the midgut and entry into the haemocoel (body cavity of the mosquito). In *Aedes albopictus* especially, CHIKV shows very efficient dissemination — in one study nearly 100 % of infected mosquitoes achieved disseminated infection rapidly (Carpenter and Clem, 2023). After dissemination into the haemocoel, CHIKV reaches the salivary glands, replicates there, and is secreted into the saliva. The mosquito then becomes capable of transmitting the virus to a vertebrate host during a subsequent blood meal (Dong et al., 2017). These mosquito species frequently feed on humans and are widespread, providing ample opportunities for virus acquisition and transmission and viral genetic adaptations (e.g., the E1-A226V mutation in CHIKV) increase infectivity and dissemination in *Aedes albopictus* and Low or absent barriers (especially dissemination barrier) in *Aedes albopictus* enable rapid spread from midgut to saliva (Viginier et al., 2023).

*Aedes aegypti* and *Aedes albopictus* possess innate immune responses that can limit CHIKV replication. The RNA interference (RNAi) pathway is the major antiviral defense mechanism identified in *Aedes aegypti* against CHIKV, acting to degrade viral RNA and reduce viral load within the mosquito (McFarlane et al., 2014). Laboratory studies have demonstrated that CHIKV can be vertically transmitted from infected female mosquitoes to their offspring. Vertical transmission of CHIKV has been detected in both *Aedes aegypti* and *Aedes albopictus* (Chompoosri et al., 2016), The eggs of *Aedes albopictus* can survive extended dry and cold periods due to diapause. Studies show that diapause eggs can remain viable even after exposure to −10°C, suggesting potential overwinter survival. However, whether CHIKV remains infectious or simply persists in a latent form within overwintering eggs remains uncertain (Thomas et al., 2012).

### 8.1 Strategies for controlling *Aedes aegypti* and *Aedes albopictus*

The invasive mosquitoes *Aedes aegypti* and *Aedes albopictus* are vectors for significant arboviral diseases including *dengue, Zika, chikungunya, and yellow fever viruses*. Even in non‑endemic countries, global travel and climate change elevate their threat. Aedes species predominantly transmit arboviruses, and their control is essential even in regions where diseases are not endemic. Prevention relies mainly on vector control, especially since vaccines may be unavailable or limited in efficacy. Integrated vector management, tailored for non‑endemic countries, is crucial.

#### 8.1.1 Chemical control and resistance issues

Organophosphates such as temephos (trade name Abate) have long been used in larval source management but face growing resistance and environmental toxicity concerns. In Sri Lanka, *Aedes aegypti* resistance to temephos has been reported, necessitating new strategies to maintain efficacy (Fernando et al., 2020). Pyrethroids (e.g., permethrin, deltamethrin) are widely applied due to high efficacy and relatively low impact on non‑target species, but resistance through target site mutations (kdr mutations) and enhanced metabolic detoxification is increasing. Moreover, alterations in cuticle thickness have been linked to reduced penetration of the insecticide in *Aedes albopictus*. These findings threaten long‑term effectiveness (Moyes et al., 2017). The World Health Organization (WHO) recommends several pyrethroids (such as alpha cypermethrin and delta methrin) for indoor residual spraying and use in bed nets, yet their continued utility is threatened by growing resistance (WHOWGroirimvGW, 2018).

#### 8.1.2 Biological and eco friendly alternatives

Bacillus thuringiensis israelensis (Bti) is environmentally safe, biologically specific, and there is no widespread resistance. It is employed globally through commercial products such as VectoBac® and Mosquito Dunks (Lacey, 2007). Agents like diflubenzuron, pyriproxyfen, and spinosad have demonstrated high efficacy even in temephos‑resistant Aedes populations and are safe for use in potable water sources (Boyer et al., 2018). Species such as Beauveria bassiana and Metarhizium anisopliae can significantly impair adult survival, fecundity, and larval development across all life stages of *Aedes* mosquitoes (Scholte et al., 2004). Copepods (e.g., Mesocyclops spp.) and other aquatic predators have been shown to substantially reduce larval densities. A study in Vietnam reported near‑complete elimination of Ae. aegypti from several communities using copepods as biological control agents (Kay and Vu, 2005). These traps entice gravid females to lay eggs and simultaneously kill adults and/or larvae without widespread pesticide use. Field trials have shown reductions of 92 % to 97 % in *Aedes* populations and corresponding drops in dengue incidence (Barrera et al., 2014).

### 8.2 Genetic and Wolbachia‑based interventions

Release of radiation‑sterilized male mosquitoes can suppress reproduction. In Guangzhou, China, combining SIT with incompatible Wolbachia infections nearly eradicated local Ae. albopictus populations (Zheng et al., 2015). Infecting mosquitoes with Wolbachia bacteria reduces their capacity to transmit *arboviruses*. Programs led by the World Mosquito Program have achieved record reductions in dengue incidence, such as in Colombia, with ongoing scale ups in other regions (Utarini et al., 2021). Companies like Oxitec have developed genetically engineered mosquitoes whose female offspring do not survive. Field releases in Brazil suppressed wild populations by up to 96 %. These methods are species specific, though repeated releases are necessary to sustain suppression (Carvalho et al., 2015).

### 8.3 Integrated control strategy for non‑endemic countries

Non‑endemic countries must emphasize proactive and multi‑layered strategies:

**Surveillance**: Regular monitoring at international entry points (airports, seaports), and checking high‑risk breeding sites, **Source reduction**: Public education campaigns to eliminate standing water sources like coolers, unused containers, and water‑storage tanks (Rahman et al., 2025). **Targeted interventions**: Deploy Bti, diflubenzuron, lethal ovitraps, and alternative larvicides selectively in areas of suspected introduction, **Rapid response**: Upon detection of *Aedes* presence, rapidly deploy SIT, Wolbachia, or genetic control measures to prevent establishment, **Resistance monitoring**: Routine assays to assess insecticide susceptibility and detect resistance trends early (Weaver and Lecuit, 2015). **Environmental and ecological caution**: Favor eco friendly, species specific methods over broad‑spectrum chemicals to protect non‑target organisms.

Effective control of *Aedes aegypti* and *Aedes albopictus* in non‑endemic countries demands a hybrid approach combining chemical, biological, genetic, and environmental methods. As traditional insecticides lose effectiveness, integrating novel, sustainable tools backed by vigilant surveillance, public education, and ecological mindfulness offers a resilient path forward. Ongoing innovation and adaptive management are essential to meet evolving challenges.

In vivo models of CHIKV infection and their contribution to understanding disease mechanisms

## 9 In vivo models of CHIKV infection and their contribution to understanding disease mechanisms

**Fig. 1:**
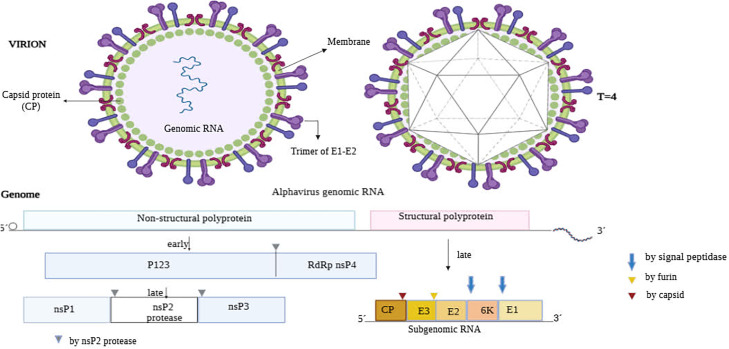
Diagram depicting the structure and genome organization of chikungunya virus (CHIKV): The upper panels illustrate the virion’s lipid envelope, trimeric glycoproteins (E1-E2), and capsid protein (CP) assembling around genomic RNA, as well as its icosahedral symmetry. The lower panels show the alphavirus genomic RNA encoding non-structural and structural polyproteins, with protease-mediated cleavage sites and the subgenomic RNA expressing the capsid, envelope proteins, and 6 K protein.

**Fig. 2:**
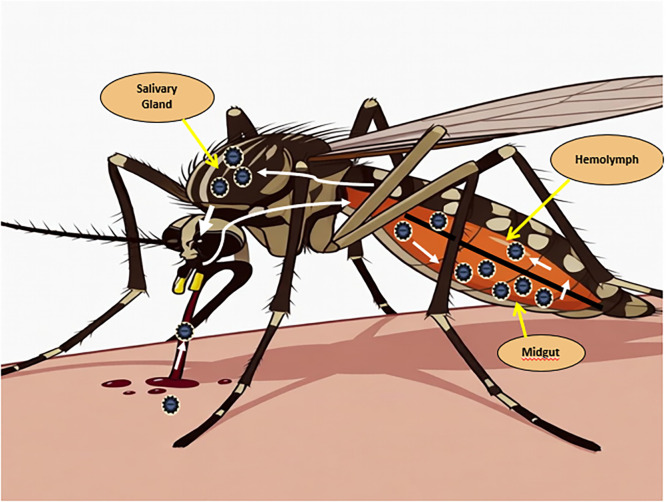
CHIKV cycle in mosquito. Mosquito feeds on virus-infected blood. The ingested infected blood travels to the mosquito’s midgut, where the virus initiates its replication. The virus enters the mosquito’s circulatory system, allowing dissemination throughout the body. The virus travels to the mosquito’s salivary glands, where it accumulates in preparation for transmission. During the next bite, the mosquito injects virus-laden saliva into a new host before blood feeding, completing the transmission cycle.

**Fig. 3:**
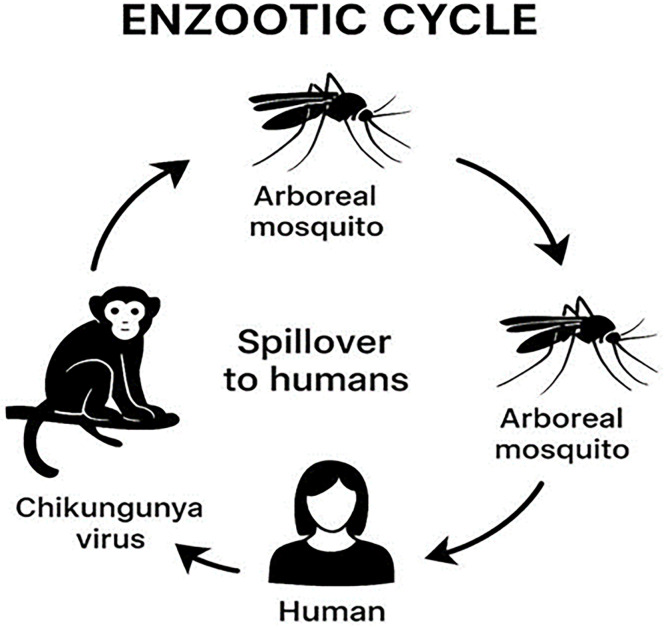
Sylvatic cycle of chikungunya virus showing transmission between arboreal mosquitoes, non-human primates, and spillover to humans.

The contribution of CHIKV infection in vivo models in understanding disease processes .The pathophysiology of CHIKV infection and the mechanisms underlying both acute and chronic symptoms of illness have been understood due in particular to in vivo animal models. The most prevalent models include those from mice and primate species (NHPs), that provide further understanding into tissue-specific disease, immunological responses, and viral tropism. CHIKV has been demonstrated to cause acute arthritis and myositis in immunocompetent and immunodeficient mice by experimental infection. These conditions are defined by viral replication in musculoskeletal tissues with significant inflammatory infiltrates, which resembles major features of human disease (Haese et al., 2016) (Fig. 1, Fig. 2, Fig. 3).

Further research on mice has demonstrated that disruption of type I interferon signaling significantly raises viral replication and illness severity, emphasizing the crucial function of innate immunity in managing CHIKV infection. Mice lacking the IFN-α/β receptor and IRF3/7 exhibit severe and frequently fatal illness, indicating that type I interferon responses have a crucial role in determining viral restriction and disease severity. In addition, immunocompetent C57BL/6 mouse models have been used to study chronic disease, showing prolonged low-level viral persistence in joint-associated tissues, cartilage damage, and periosteal bone changes, which parallel chronic arthralgia observed in patients (Carlos Ralph Batista Lins CdSC, 2022). Nonhuman primate models, particularly cynomolgus and rhesus macaques, more closely reproduce the clinical, virological, and immunopathological features of human chikungunya disease. CHIKV infection in cynomolgus macaques results in acute viremia, fever, and involvement of joints, muscles, lymphoid organs, and liver, providing a robust platform for studying systemic disease and viral dissemination. Importantly, NHP studies have identified macrophages as major cellular reservoirs during late stages of infection, demonstrating long-term viral persistence in tissue macrophages, which provides a mechanistic explanation for prolonged inflammation and chronic musculoskeletal symptoms in humans (Labadie et al., 2010).

Recent in vivo proteomic and multi-organ analyses in rodent and NHP models have further expanded understanding of CHIKV pathogenesis beyond joints and muscles. These studies revealed dysregulated immune and metabolic pathways across multiple organs and identified host factors such as ISG15, RIG-I, S100A8/A9, and complement component C3 as contributors to CHIKV-induced inflammation and tissue damage, underscoring the systemic nature of CHIKV disease. Collectively, these in vivo studies demonstrate that animal models are essential for defining viral tropism, immune-mediated pathology, and mechanisms of viral persistence, thereby providing critical insights into both acute and chronic manifestations of chikungunya and informing the development of targeted therapeutic and vaccine strategies (Lin et al., 2025). In addition, a mouse model study focusing on the East/Central/South African (ECSA) genotype provided important insights into the relationship between viral pathogenicity, interferon responses, and host metabolic activity. Using type I interferon receptor–deficient (A129) and type I/II interferon receptor–deficient (AG129) mice, infection with the ECSA M-30 strain resulted in significantly higher and more acute fatality compared with the prototype African S-27 strain following low-dose challenge. M-30-infected mice exhibited markedly higher viral loads in both central nervous system and peripheral organs, together with increased IFN-γ responses in the brain, indicating enhanced viral dissemination and neurotropism (Ngwe Tun et al., 2020). Notably, M-30-infected mice did not develop hypophagia or body weight loss, in contrast to S-27-infected animals, suggesting atypical metabolic responses during severe disease. The authors proposed that the increased pathogenic potential of the ECSA M-30 strain is associated with virus proliferation, resistance to type II interferon–mediated control, and altered host metabolic activity, thereby highlighting metabolic dysregulation as a previously underappreciated component of CHIKV pathogenesis in vivo (Lin et al., 2025) (Table 1, Table 2, Table 3).

**Table 1:** Impacts of mutations on the virus adaptation (Shandhi et al., 2025).

Mutation	Target of mutation	Impact
E1-A226V	E1 Glycoprotein	Most famous adaptive mutation. It involves the substitution of Alanine (A) with Valine (V) at amino acid position 226. This mutation significantly enhances the virus's fitness and transmission efficiency in the *Aedes albopictus* mosquito (Asian tiger mosquito), allowing the virus to spread rapidly in regions where this mosquito species predominates (e.g., in parts of Europe and Asia).
E2-L210Q	E2 Glycoprotein	Often observed in strains that also carry the E1-A226V mutation, this substitution (Leucine to Glutamine) also increases the virus's ability to infect and disseminate within *Aedes albopictus*. It represents a further step in the virus's multi-step adaptation to this vector.
E1-K211E / E2-V264A	E1 and E2 Glycoproteins	These co-mutations have been associated with fitness advantages in the primary vector, *Aedes aegypti* (yellow fever mosquito), suggesting a vector-switching or maintenance strategy in areas where *Ae. aegypti* is the main vector.
Non-Structural Protein (nsP) Mutations	Non-Structural Proteins	Mutations in genes coding for non-structural proteins (nsP1–4) can influence the viral replication rate, pathogenicity, or, in some cases, lead to resistance against antiviral drugs (K291R (Lysine to Arginine at position 291)) like *Favipiravir*.

**Table 2:** Summary of Chikungunya Virus Epidemiology, Prevalence, and Mortality in Selected African Countries.

Country/ Territory	Region	Activity periods	Estimated prevalence rate	Case fatality rate (CFR)
Tanania	East Africa	1952–1953	7.8 % (CHIKV positive rate among febrile patients in Northern Tanzania, specific study) (Faustine et al., 2017)	Low (Generally <0.1 %) (Silva and Dermody, 2017)
Kenya	East Africa	2004	12.2 % (Pooled prevalence in East Africa systematic review) 64.9 % (Seropositivity in a study of adults in 2007) (Ali Mude et al., 2024, Prevalence and factors 2007)	Low (Consistent with global average, usually <0.1 %) (Silva and Dermody, 2017)
Djibouti	East Africa	2019–2020	50.4 % (Pooled prevalence in East Africa systematic review) 12.3 % (Attack Rate of suspected cases in 2019–2020 outbreak) (Ali Mude et al., 2024, Javelle et al., 2023)	Not explicitly reported (Outbreak data focused on attack rate, not CFR)
DR Congo (DRC)	Central Africa	1999–2000	49.7 % (RT–qPCR positive rate among patients tested during the 2019 outbreak), 83.2 % (Acute infection rate in clinical suspects during 2019 Matadi outbreak) (Selhorst et al., 2020, De Weggheleire et al., 2021)	Low (Mortality is seldomly reported but low; overall lethality is 1 %−2 % in acute cases for certain outbreaks, generally for high-risk groups) (De Weggheleire et al., 2021)
Cameroon	Central Africa	2006	0.5 % (RT-PCR positive rate among febrile patients in a 2022 study) 18.4 % to 21.7 % (IgM seroprevalence rate depending on the region) (Nana-Ndjangwo et al., 2022)	Very Low (Generally <1 per 1000 cases) (Silva and Dermody, 2017)
La Réunion	Indian Ocean (Africa)	2005–2006	∼32 % (Estimated population affected during the 2005–2006 outbreak, resulting in ∼250,000 cases) (Pastorino et al., 2004)	0.34 % (During the 2005–2006 epidemic, resulting in 237 deaths) (Pastorino et al., 2004)

**Table 3:** Annual Case Reports (2021–2024) (Schwameis et al., 2016).

Year	Total Suspected chikungunya Cases (All India)	Total Confirmed chikungunya Cases (All India)
2021	119,070	12,254
2022	148,587	10,724
2023	93,455	9072
2024 (partial Data, to Aug)	30,661	13,858

## 10 Recent global outbreaks of chikungunya virus (2024–2025)

Chikungunya virus (CHIKV) infection has been significantly underreported in various regions worldwide; however, it experienced an extraordinary resurgence between 2024 and 2025 (Silva et al., 2019). The World Health Organization (WHO) documented more than 1.6 million suspected cases, including what has been identified as the largest local outbreak in China, which alone accounted for over 160,000 laboratory-confirmed cases. By September 2025, Africa reported 2197 suspected cases and 108 confirmed cases of CHIKV infection. Furthermore, as of October 3, 2025, Europe recorded a total of 56,456 CHIKV cases, with 40 fatalities, across four countries and regions: France, Italy, Reunion, and Mayotte. In Asia, the Western Pacific subregion of East Asia, Southeast Asia, Central Asia, and South Asia show much more instances of CHIKV (Ribeiro Dos Santos et al., 2025). In countries like Bangladesh, Indonesia, and Pakistan, the prevalence of CHIKV cases has risen considerably during the past two years.China's Guangdong province recorded more than 16,000 laboratory-confirmed cases as of September 27, 2025, making it the country's largest-ever chikungunya fever outbreak. By November 15, an additional 48 local cases were identified within this province. The reported cases in Guangdong have spread across 21 cities, with the majority occurring in Foshan (10,040 cases), Jiangmen (5223 cases), Guangzhou (590 cases), Shenzhen (140 cases), Zhanjiang (112 cases), Zhuhai (60 cases), and Zhongshan (54 cases). Notably, individuals aged 18 to 45 years comprised the largest demographic among the reported cases (Chikungunya, 2025).

Global surveillance data collected up to the third quarter of 2025 indicate a substantial increase in CHIKV circulation, with reports of over 445,000 suspected and confirmed cases and 155 associated fatalities globally across at least 40 countries (Disease Outbreak News, 2025).

### 10.1 Africa

CHIKV Since its initial identification in Tanzania in the early 1950s, the virus has been the cause of recurring outbreaks that pose a significant public health threat, especially in resource-limited settings (Silva and Dermody, 2017, Chinedu Eneh et al., 2023, Kayange et al., 2023). The global expansion of CHIKV in the 21st century is established in its African ecology, which is characterized by complex enzootic (sylvatic) and epidemic (urban) transmission cycles (Zeller et al., 2016, Anjos et al., 2023).

#### 10.1.1 Historical context and transmission dynamics

The first documented isolation of CHIKV occurred during an outbreak in the Makonde area of southern Tanzania between 1952 and 1953 (Chinedu Eneh et al., 2023, Kayange et al., 2023, Russo et al., 2020). For decades thereafter, CHIKV circulation was reported sporadically across the continent, particularly in Central, East, and Southern Africa (Zeller et al., 2016, Russo et al., 2020). A major epidemiological shift was noted in 2004, beginning with a large outbreak on the coast of Kenya. This event marked the beginning of an accelerated geographic expansion, spreading rapidly to the islands of the Indian Ocean (such as La Réunion and Mayotte) and subsequently increasing a global pandemic (Zeller et al., 2016, Pialoux et al., 2007).

CHIKV strains in Africa primarily belong to the East/Central/South African (ECSA) lineage and the West African lineage (Anjos et al., 2023). The ECSA lineage is associated with the large, explosive outbreaks in East Africa and the Indian Ocean. A critical finding during the 2005–2006 La Réunion outbreak was the A226V point mutation in the E1 gene, which significantly increased the virus's fitness and transmission efficiency in the Aedes albopictus mosquito, a vector common in urban settings (Pialoux et al., 2007). In West Africa, transmission dynamics are often characterized by sporadic emergence events resulting from enzootic spillover from a sylvan cycle involving non-human primates (Zeller et al., 2016, Anjos et al., 2023). Despite recent advances, the true burden of CHIKV in many African nations is underestimated due to a combination of under-diagnosis, under-reporting, and the virus co-circulating with other febrile illnesses like Dengue and Malaria (Chinedu Eneh et al., 2023, Kayange et al., 2023, Russo et al., 2020).Recent systematic reviews highlight significant prevalence rates, especially in East Africa (Ali Mude et al., 2024).

#### 10.1.2 Factors contributing to chikungunya virus endemicity in Africa

The continent of Africa is recognized as the original ecological niche for the chikungunya virus (CHIKV), where the virus is maintained in a state of endemicity through complex ecological and viral mechanisms, differentiating its circulation from explosive epidemic patterns seen in other parts of the world (Silva and Dermody, 2017). The sustained presence of CHIKV is fundamentally fixed in the stability of its sylvatic (enzootic) transmission cycle and subsequent spillovers into human populations (Zeller et al., 2016, Ramphal et al., 2024).

#### 10.1.3 The sylvatic cycle and non-human primate reservoir

A primary driver of African endemicity is the established enzootic cycle or sylvatic cycle, CHIKV is maintained in nature through alternating transmission between forest-dwelling mosquito vectors and nonhuman vertebrate hosts (primarily nonhuman primates), independent of sustained human involvement) (Eastwood et al., 2017). Involving forest-dwelling mosquitoes and non-human primates (NHPs), particularly across West and Central African regions (Zeller et al., 2016, Russo et al., 2020, Chikungunya, 2025). The virus is known to be maintained in this jungle cycle, with studies in Uganda dating back to the 1950s detecting CHIKV viremia and antibodies in sentinel monkeys (Zeller et al., 2016). This reservoir ensures the continuous presence of the virus, allowing it to re-emerge periodically. Outbreaks in certain regions, such as Senegal, have demonstrated a cyclical pattern of emergence (every 4–7 years), which is theorized to align with the renewal of susceptible, non-immune NHP populations (Zeller et al., 2016, Chikungunya, 2025). This repeated enzootic spillover into humans prevents the virus from being completely cleared from the continent (Ramphal et al., 2024).

#### 10.1.4 Presence of diverse and competent mosquito vectors

Africa harbors a wide array of competent mosquito vectors necessary to sustain both the sylvatic and urban transmission cycles. Sylvatic Vectors: Forest-dwelling Aedes species, such as *A. africanus* in East Africa and *A. furcifer* and *A. taylori* in West Africa, maintain the enzootic cycle (Zeller et al., 2016).Urban Vectors: The peridomestic mosquitoes, primarily *Aedes aegypti* and the invasive *Aedes albopictus,* drive the epidemic cycle when the virus spills over into human populations (Russo et al., 2020). The increasing spread of Aedes albopictus, coupled with the evolution of the E1-A226V mutation in the East/Central/South African (ECSA) lineage, has enhanced the virus's fitness for transmission in urban settings across the continent and surrounding islands (Nana-Ndjangwo et al., 2022, Pastorino et al., 2004).

#### 10.1.5 Under-diagnosis and persistent undetected circulation

A significant epidemiological factor is the prevalent issue of under-diagnosis and under-reporting CHIKV infection can be asymptomatic in up to 80 % of cases, which means a large portion of circulation remains undetected by passive surveillance systems (Chikungunya, 2025). In symptomatic patients, the illness often resembles other co-circulating febrile diseases like Malaria and Dengue fever, leading to frequent misdiagnosis in low-resource settings (De Weggheleire et al., 2021). This lack of standardized surveillance and clinical confusion means that endemic circulation continues largely unchecked, allowing the virus to persist in the human and vector populations at low levels until conditions (such as vector population booms or the introduction of a new, highly susceptible human population) trigger an outbreak (Russo et al., 2020, Chikungunya, 2025).

The endemic nature of CHIKV in Africa is a result of a stable, ancient sylvatic cycle serving as a permanent reservoir, a diverse ecology of competent Aedes vectors facilitating spillover, and systemic challenges in surveillance and diagnosis that mask the true extent of the virus's persistent circulation (Zeller et al., 2016, Russo et al., 2020, Ramphal et al., 2024).

### 10.2 Asia

The Asian emergence of CHIKV marked the beginning of its global spread outside of its ancestral African enzootic focus, establishing a persistent urban transmission cycle distinct from the sylvan cycles of Africa (Tsetsarkin et al., 2011). While retrospective serological evidence suggests human CHIKV infection may have occurred in India as early as 1954 (Wimalasiri-Yapa et al., 2019), the first laboratory-confirmed and extensively documented urban epidemic of chikungunya fever in Asia was reported in Bangkok, Thailand, in 1958 (Wimalasiri-Yapa et al., 2019, Krambrich et al., 2024, Thavara et al., 2009).

#### 10.2.1 High burden and endemicity of CHIKV in India

India consistently reports one of the highest cases of CHIKV cases globally and bears a substantial long-term burden from the disease, primarily due to a convergence of favorable environmental conditions, demographic density, and viral adaptations (Kang et al., 2025). The number of suspected and confirmed CHIKV cases remains high, with significant year-to-year variation reflecting the cyclical, epidemic nature of the virus (NRaGP, 2024).

A systematic review and meta-analysis found the overall mortality rate among patients with CHIKV infection to be approximately 0.3 % (95 % CI: 0.1 %−0.7 %), the table reflects the acute nature of the disease, which is generally self-limiting but with high morbidity (Rama et al., 2024).

### 10.3 Europe

In Europe, the predominant vector is *Aedes albopictus* (the Asian tiger mosquito), which has become widely established across Mediterranean and temperate regions. Laboratory and field-derived vector competence studies confirm that *Ae. albopictus* populations in Europe are capable of acquiring CHIKV infections, supporting viral dissemination to salivary glands, and transmitting the virus via bites under favorable temperature conditions (Viginier et al., 2023). Although CHIKV is not endemic in Europe, imported and sporadic cases have been reported due to favorable climates in Mediterranean areas (ANON, 2025a). French overseas territories experienced intense outbreaks in 2024–2025. La Réunion reported over 47,500 cases and 12 deaths by mid-2025 (WHOWCEU-J, 2025). Mayotte reported 116 cases with both imported and local transmissions (WHOWCEU-J, 2025, Pan American Health Organization (PAHO) 2025). Mainland France recorded approximately 800 imported CHIKV cases since May 2025, while Italy confirmed its first locally transmitted case in the same period (ANON, 2025a). Enhanced vector monitoring and public education campaigns have been initiated to prevent further spread. Vector control for CHIKV focuses principally on managing *Aedes albopictus* through harmonised surveillance, habitat reduction, and targeted interventions, the AIMSurv project has unified mosquito monitoring across many countries using standard protocols to detect invasive *Aedes* species early (Miranda et al., 2022). The ECDC likewise issues technical guidelines for surveillance, recommending trap deployment, larval monitoring, and data sharing to support decision-making. Environmental management removal of standing water, covering containers, improving drainage is promoted as a core prevention measure (Vector, 2017).

### 10.4 Americas

In the United States, surveillance data indicate that most documented infections since the virus’s introduction to the Americas have been travel-related, while local transmission has been sporadic and geographically limited to a small number of states and U.S. territories (notably Florida, Texas, Puerto Rico and the U.S. Virgin Islands during the 2014–2019 epidemic period); routine arboviral surveillance and retrospective analyses emphasize continued vulnerability to re-introduction because of widespread presence of *Aedes* vectors and ongoing international travel (Chikungunya in the United, 2025, Duncan and Eppes, 2025).

The Americas have been a major hotspot for CHIKV activity in recent years, with a high number of reported cases and associated deaths, particularly South America, has high burden of recent CHIKV activity. In 2025, 14 countries in the Americas reported 212,029 suspected cases and 110 deaths, with over 97 % from South America. Brazil, Bolivia, and Paraguay were especially impacted (Pan American Health Organization (PAHO), 2025; ANON, 2024). The re-emerging is linked to increased urban mosquito habitats and limited vector control resources (ANON, 2025b). Localized outbreaks in the Caribbean, including Jamaica, the Dominican Republic, and Puerto Rico, have been reported in 2024–2025. Pan American Health Organization (PAHO) has advised stronger vector surveillance and community engagement to curb transmission (Pan American Health Organization (PAHO) 2025).

### 10.5 Australia

CHIKV is currently not endemic to Australia, and all documented human cases have been imported from abroad rather than resulting from local transmission. According to the Victorian Health Department, no locally acquired cases have ever been confirmed in Australia, and every reported case to date has been traced to overseas exposure. In both the United States and Australia, the principal competent vectors for chikungunya virus are *Aedes aegypti* and *Aedes albopictus* (Chikungunya in the United, 2025, Hugo et al., 2019). Oceania has seen limited CHIKV activity recently, but remains at risk due to proximity to Southeast Asia and the Pacific Islands where vectors are abundant (WHOWCEU-J, 2025). Regional health authorities maintain vigilance through surveillance and public awareness campaigns (WHOWCEU-J, 2025).

The recent resurgence and global spread of CHIKV between 2024 and 2025 highlight significant public health challenges. Factors such as urbanization, climate change, and global travel facilitate vector expansion and viral transmission (Pialoux et al., 2007). Continuous surveillance, improved vector control, and international cooperation remain critical to mitigate chikungunya 's impact worldwide (WHOWCEU-J, 2025, Pan American Health Organization (PAHO) 2025).

This review provides a comprehensive update on chikungunya virus up to mid-2025. It integrates the latest epidemiology, vaccine developments, and genetic diversity, focusing on mutations affecting transmission. The article critically evaluates diagnostic tools, details pathogenesis and immune evasion, and explores virus-mosquito interactions, including transmission dynamics. It presents integrated vector control strategies and a structured regional outbreak analysis, while offering a balanced review of vaccine candidates, including recent safety and regulatory updates.

## 11 Conclusion

CHIKV causes substantial illness, is spreading into new regions, and continues to circulate through Aedes mosquitoes, making it a major global public health issue. New vaccines, especially those targeting the viral envelope proteins, offer real promise for protecting populations and lowering disease burden, but they cannot solve the problem on their own.​ To truly curb mosquito numbers and prevent future outbreaks, particularly as climate change expands suitable habitats, public health programs must combine chemical, biological, and genetic vector-control tools rather than relying on a single method. Ongoing progress will also depend on deeper research into CHIKV pathogenesis, host immune responses, and viral evolution, together with stronger surveillance systems and tightly coordinated public health actions at national and international levels. Only this comprehensive strategy can meaningfully protect vulnerable communities and lessen the long-term health and socioeconomic burden associated with chikungunya worldwide.

## Data availability

No data was used for the research described in the article.

## CRediT authorship contribution statement

**Morvarid Hamrahjoo:** Writing – review & editing, Writing – original draft. **Faezeh Shams:** Writing – review & editing. **Nastaran Saadat:** Writing – review & editing. **Shayan Marhamati:** Writing – review & editing. **Ali Teimoori:** Writing – review & editing, Supervision, Funding acquisition.

## Declaration of competing interest

The authors declare no conflict of interest.
